# Assessment of Primary Human Liver Cancer Cells by Artificial Intelligence-Assisted Raman Spectroscopy

**DOI:** 10.3390/cells12222645

**Published:** 2023-11-17

**Authors:** Concetta Esposito, Mohammed Janneh, Sara Spaziani, Vincenzo Calcagno, Mario Luca Bernardi, Martina Iammarino, Chiara Verdone, Maria Tagliamonte, Luigi Buonaguro, Marco Pisco, Lerina Aversano, Andrea Cusano

**Affiliations:** 1 Optoelectronic Division-Engineering Department, University of Sannio, 82100 Benevento, Italy; 2 Centro Regionale Information Communication Technology (CeRICT Scrl), 82100 Benevento, Italy; bernardi@unisannio.it (M.L.B.); l.buonaguro@istitutotumori.na.it (L.B.);; 3 Informatics Group, Engineering Department, University of Sannio, 82100 Benevento, Italy; 4 National Cancer Institute-IRCCS “Pascale”, Via Mariano Semmola, 52, 80131 Napoli, Italy

**Keywords:** liver cancer cells, machine learning, neural networks, Raman spectroscopy

## Abstract

We investigated the possibility of using Raman spectroscopy assisted by artificial intelligence methods to identify liver cancer cells and distinguish them from their Non-Tumor counterpart. To this aim, primary liver cells (40 Tumor and 40 Non-Tumor cells) obtained from resected hepatocellular carcinoma (HCC) tumor tissue and the adjacent non-tumor area (negative control) were analyzed by Raman micro-spectroscopy. Preliminarily, the cells were analyzed morphologically and spectrally. Then, three machine learning approaches, including multivariate models and neural networks, were simultaneously investigated and successfully used to analyze the cells’ Raman data. The results clearly demonstrate the effectiveness of artificial intelligence (AI)-assisted Raman spectroscopy for Tumor cell classification and prediction with an accuracy of nearly 90% of correct predictions on a single spectrum.

## 1. Introduction

Liver cancer remains a global health challenge and its incidence is increasing worldwide [1]. It is estimated that liver cancer will affect more than 1 million people annually by 2025 [2]. Epidemiological data also indicate an increase in liver cancer-related deaths, ranking this malignant disease as the third leading cause of death worldwide [3]. Moreover, liver tumors have been predicted to be among the leading causes of death by 2030 [4].

Liver cancer is defined as a heterogeneous group of tumors characterized by different histological, molecular, and clinical features [5]. The most common form of primary liver cancer in adults is hepatocellular carcinoma (HCC), with an incidence rate of 90% [2].

In clinical practice, the traditional diagnosis of HCC relies on various imaging methods such as ultrasonography (US), computed tomography (CT), or magnetic resonance imaging (MRI). However, the unsatisfactory sensitivity, need for standardization, operator-dependent results of US, high cost, and time-consuming procedures for CT and MRI make the diagnosis of HCC difficult [6]. In cases where there are no clear CT/MRI findings, a liver biopsy is performed.

Tissue samples taken during a liver biopsy must be sent to an analytical laboratory for histopathologic diagnosis or cytological analyses, which are performed at the tissue and cellular levels, respectively. The gold standard protocol requires that the collected specimens undergo various preparation steps (dehydration, fixation, waxing, staining, slicing, and mounting), which require labor and time that typically ranges from 10 h to 3 days [7]. In addition, the accuracy of pathological diagnosis is influenced by the subjective factors of pathologists [8].

Both histological and cytological analyses provide important information for diagnosis and prognosis. Indeed, it is necessary to study cells at both the multicellular and single-cell levels to provide scientists with a balanced and comprehensive picture. Cell-to-cell variation is a natural feature of both healthy and diseased tissues or multicellular organisms that pose important challenges in drug discovery, diagnostics, and prognostics. Statistical analysis of tissue and related whole-cell populations provides an average response across the entire population and is not fully representative of each cell, including rare variants. Therefore, the population average may mask the response of an individual cell. This heterogeneity poses a major challenge when attempting to develop accurate disease models or to elucidate patient response to specific therapies.

Therefore, a technological approach that allows rapid and “intelligent” analysis and classification of cells or tissues from tissue biopsies would be of utmost importance for improving the diagnosis and characterization of malignant cells.

In this regard, Raman spectroscopy (RS) has emerged as a powerful spectroscopic technique for clinical oncology applications because of its label-free, noninvasive, nondestructive, and rapid nature. The Raman spectrum, indeed, represents the “molecular fingerprint” of a biological sample, and the Raman bands (i.e., the peaks in the spectrum) are associated with specific chemical information about the cellular components (i.e., lipids, proteins, nucleic acids, and carbohydrates). RS has been shown to have high sensitivity and molecular specificity in studying the altered biochemical composition in tumor samples compared to its non-tumor counterpart [9].

Actually, the use of RS in cancer diagnostics is limited by the complexity of the elaboration of the Raman data for identifying vibrational fingerprints, which are useful for distinguishing between oncological and non-pathological samples [10]. Nonetheless, the use of artificially intelligent systems could significantly contribute to appreciating statistically relevant tiny differences in the spectra and support prognostic and diagnostic stages through accurate and in-depth cellular analysis.

Indeed, great efforts have been made using Artificial Intelligence (AI) methods to identify and analyze a characteristic spectral pattern for cell classification. In particular, there is scientific evidence for the application of RS in combination with machine learning approaches to classify different tumor cell types as well as to discriminate between cancer and non-cancer cells [11,12,13]. Notably, most machine-learning and Raman spectroscopy-based methods for distinguishing and classifying different human liver cancer cells and non-cancer cells used immortalized cell lines [14,15]. Although human liver cell lines are commonly used as a model for liver cancer, they represent a homogeneous cell population that lacks some liver-specific functions, has an altered genome, and exhibits atypical cell behavior compared to primary cells [16]. This leads to modeling machine learning methods that suddenly fail when applied to real classifications. On the other hand, the cultivation of primary cells is also not optimal, as only a limited number of cell cycles are observed, leading to a progressive loss of morphological, phenotypic, and functional cellular features [17]. Therefore, we focused our studies on uncultured primary human liver cancer and non-cancer cells, which represent the cell systems that better correspond to the real characteristics of the liver under pathological and physiological conditions, respectively. To note, very recently, Huang et al. investigated human samples of liver tissue using Raman spectroscopy and found remarkable differences between tumor and non-tumor tissues [18]. Nonetheless, an investigation on the use of AI-assisted Raman spectroscopy to recognize primary human liver cancer cells has never been reported.

In this study, we report the first Raman spectral analysis based on characterization and differentiation between uncultured primary human liver cancer and non-cancer cells belonging to an HCC patient. Raman analysis of the two primary cell samples was performed to identify the molecular spectral fingerprint reflecting a different biochemical composition of the two samples.

Two basic machine learning models based on Linear Discriminant Analysis (LDA) were explored to further automate and accelerate data analysis to reveal hidden patterns correlated with pathology.

LDA is a statistical method used to find linear combinations of features that best separate classes of data. PCA is a dimensionality reduction technique used to decrease the dimensionality of the data by projecting it onto a subspace with a lower dimensionality subspace while preserving as much variance as possible. Therefore, we used an LDA model applied directly to the Raman spectra, namely the “Hyper-parameter tuned LDA”, and an LDA model applied to the PCA components, namely the “PCA-LDA” model, to extract the most informative features and feed them into a classifier.

Alternatively, we developed a neural network-based ensemble based on the combination of convolutional and recurrent neural networks. Convolutional Neural Networks (CNNs) are particularly effective at extracting relevant features for recognizing complex patterns [19], such as Raman spectra, while recurrent networks based on Long-Short Term Memory cells (LSTMs) are effective at learning and detecting relationships in sequences [20]. The neural network ensemble, namely the “CNN-LSTM” model, is proposed as an additional model for recognizing and rapidly classifying specific Raman fingerprints of Non-Tumor and Tumor cells, to assist clinicians in diagnosing HCC liver cancer.

The ability to discriminate between cancer and non-cancer cells was assessed using three artificial intelligence models to blindly classify unknown cells. The results obtained outline the importance of combining Raman spectroscopy and machine learning to develop a diagnostic tool to improve HCC diagnosis.

## 2. Materials and Methods

### 2.1. Sample Collection and Preparation

Primary human liver cells were provided by the National Cancer Institute “Fondazione Pascale” from tumoral and adjacent non-tumoral tissues from the resected liver of an HCC patient. The protocol was approved by the Ethics Committee (approval no. 421/13). Only one patient was involved in this study. After resection, both tissues were dissociated into single-cell suspensions using the GentleMACS dissociator (GentleMACS, Miltenyi Biotec, Bergisch Gladbach, Germany). Afterward, the fresh uncultured Non-Tumor and Tumor cell populations were seeded onto CaF_2_ slides. Subsequently, the samples were fixed with 2% paraformaldehyde (Sigma-Aldrich, Saint Louis, MO, USA). Moreover, two additional samples were prepared using the same procedure, mixing different proportions of cancer and non-cancer cells. Specifically, the first additional sample contained 20/80% Non-Tumor/Tumor cells and the second consisted of a 40/60% Non-Tumor/Tumor cell fraction (namely, MIX1 and MIX2, respectively). Before Raman measurements, samples were washed three times with a solution of H_2_O MilliQ and 0.02% sodium azide (Sigma-Aldrich) and air dried. After completion of Raman analysis, samples were stored at +4 °C in a buffer medium.

### 2.2. Raman Spectroscopy

Raman measurements were performed using a LabRAM HR Nano (Horiba Jobin Yvon S.A.S., Longjumeau Cedex, France). The system was equipped with a frequency-doubled Nd:YAG laser operating at an excitation wavelength of 532 nm and providing a maximum output power of approximately 30 mW. All measurements were performed with an output power of approximately 3 mW. A 100× air (Olympus, Tokyo, Japan) objective with a numerical aperture (NA) of 0.90 was used. By using these settings, the calculated diameter of the laser spot is 721 nm. In the HORIBA system, the collected light is focused with an adjustable pinhole, which was set at 50 µm for all experiments. The backscattered light was spectrally separated using 300 lines per mm grating and detected with a CCD Camera (Syncerity CCD Deep Cooled Camera, cooled to −60 °C). Raman spectra were recorded in a range of 600–1800 cm^−1^ (fingerprint region, FP). Raman spectra were acquired by focusing on the cell nucleus after visual inspection of the bright-field image; at least 5 spectra were acquired for each cell. The integration time and number of accumulations per Raman spectrum were 60.0 s and 2, respectively.

### 2.3. Raman Data Pre-Processing

**Figure 1:**
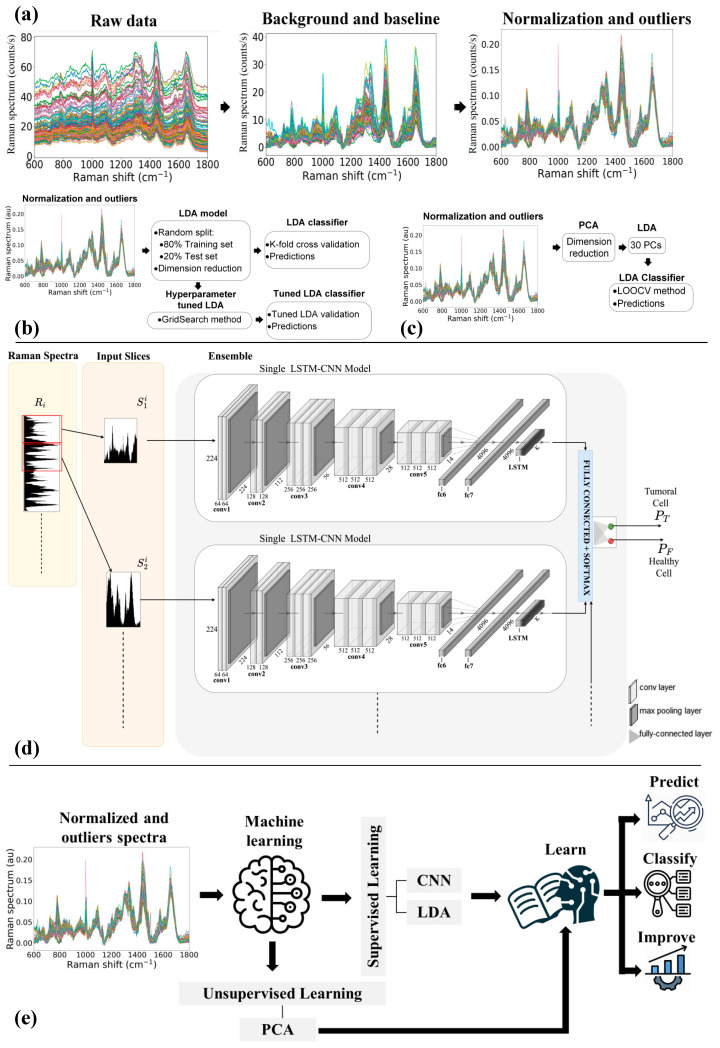
(**a**) Raman spectra preprocessing steps. Architecture of the classifier based on (**b**) LDA and Tuned-LDA model, (**c**) PCA-LDA model, and (**d**) Convolutional and Recurrent Neural Networks trained using sliding window on Raman Spectra. (**e**) Schematic of the machine learning models used for the blind predictions.

Prior to the application of statistical/chemometric methods, a pre-processing procedure is required to correct any contributions that corrupt the Raman spectra [21]. To align the spectra, the system was calibrated daily to the spectral line of crystalline silicon at 520.7 cm^−1^. For background subtraction, a spectrum was recorded on the surface of the CaF_2_ substrate and subtracted from all acquired spectra. To remove the fluorescence contribution, the baseline was corrected by applying a polynomial fitting (order 3) [22]. Finally, to compare the intensities, all spectra were scaled by applying vector normalization [23]. Partial least square (PLS) regression in combination with Q-residual and Hotelling’s T-squared and a confidence level of 95% were used as metrics to identify outliers in the dataset. In Figure 1a, the preprocessing steps and their effect on the spectra are briefly highlighted.

### 2.4. Machine Learning

Preliminarily Unsupervised Multivariate Analysis (MVA) was used to classify the cells’ spectra. Specifically, PCA was applied as an unsupervised method to identify relevant differences between Non-Tumor and Tumor cells and reduce data dimensionality.

In addition, LDA was applied as a supervised method to discriminate the data and maximize the variance between the two groups. On the other hand, the predictive performance of the model was evaluated with a learning curve [24,25] based on k-fold cross-validation. By setting k = 5, k-fold cross-validation uses 4 folds (k-1) as training datasets and 1 fold for the test dataset, and the test accuracy is calculated after each k-iteration. The average of all results is then used to estimate the performance of the model built on the entire data. To overcome the overfitting, the LDA model was optimized using two different approaches [25].

#### 2.4.1. Hyper-Parameter-Tuned LDA 

First, LDA was optimized with a Hyper-parameter tuning based on a grid search method that performs a full search over a given subset of the Hyper-parameter space and selects the useful features for training the algorithm [26,27]. To test the predictive ability of the LDA model optimized for the Hyper-parameters, k-fold cross-validation (k = 5) was again used. Figure 1b resumes the main steps involved in the development of the Hyper-parameter tuned LDA model. The confusion matrix was used to evaluate the accuracy, specificity, and sensitivity of the LDA and optimized LDA methods (see Supporting Information for the used metrics definition).

#### 2.4.2. PCA-LDA

The LDA was alternatively optimized by feeding it with the components obtained from PCA, as schematically shown in Figure 1c. In this case, Leave-One-Out-Cross-Validation (LOOCV) was performed to avoid under- or over-fitting due to inappropriate selection of components and to determine the prediction of the error rate associated with the model; accordingly, the first 30 PCs were selected as input to the LDA.

Data manipulation and machine learning were performed using Python and Origin 2018 (OriginLab, Northampton, MA, USA).

#### 2.4.3. LSTM/CNN-Based Classifiers

The CNN-LSTM model combines CNN and LSTM layers to extract features from input data and provide sequence prediction. This model is widely used for activity recognition, image labeling, and video labeling that require visual time series prediction and text annotation generation. The basic architecture of the CNN-LSTM network, including the input layer, visual feature extraction, sequence learning, and output layer, is shown in Figure 1d.

The proposed architecture consists of 25 or 56 layers, including an input layer, 22 or 50 CNN layers wrapped with time-distributed layers, an LSTM layer, 2 or 5 dense layers, a dropout layer, and an output layer.

This architecture is suitable for the complex challenges of analyzing sequences within visual data. The blend of CNN and LSTM layers allows it to exhibit high performances in tasks that involve extracting intricate visual features and providing accurate sequence classification.

The motivations behind its structure lie in the following peculiar aspects:

- Feature Extraction Expertise: The CNN component of this model is adept at capturing high-level visual features from input data, making it exceptionally suitable for tasks that demand an in-depth understanding of visual content.
- Sequential Understanding: The LSTM layer, known for its exceptional ability to model sequential data and grasp the dynamics of a sequence, complements the CNN layer’s feature extraction by capturing the temporal dependencies in the data.
- Adaptability: The proposed architecture can be tailored to specific needs, with two configurations available, one with 25 layers and the other with 56 layers. This flexibility allows for fine-tuning to the demands of the task at hand.
- Robustness and Precision: The presence of layers for visual feature extraction, sequence learning, and output, as well as the inclusion of dropout layers to prevent overfitting, have been inserted to obtain precise and reliable results, even in the face of noisy or complex data.

While the overall structure has been conceived based on the above consideration and experimented with thereafter, the specific layers’ sizing and parametrization were performed using hyper-parameter optimization (HPO) based on the well-known Tree-Structured Parzen Estimator (TPE) approach [28] implemented in a framework like Optuna or Hyperopt.

We ensured that each Raman spectrum was transformed into the appropriate shape before constructing the model by using a sliding window process to extract windows of equal length for each Raman sequence.

Specifically, to feed an ensemble of CNN-based pretrained networks, each input Raman spectrum is segmented using windows of a fixed size so as to generate $k_{s}$ slices from each spectrum (referred to as $S_{j}^{i}$ where i identifies the spectrum and $j\in \{1,\ldots ,k_{s}\}$ is the number of the input channel in the ensemble). Each segment is fed as the input to one of the channels of the ensemble, which are jointly trained.

Once all data were properly vectorized, feature extraction was performed using three convolutional layers that automatically extracted features from the input sequences using the ReLu activation function, using 64, 128, and 256 filters in these convolutional layers. The height of the kernel was set to 6 and the width of the kernel was set to 4 for the convolution operation. We wrapped the convolutional layers in a time-distributed wrapper to transform the input data by adding an extra dimension at the end. We used a flattened layer to concatenate all the extracted features and pass them to the LSTM layer. Then, a 100-unit LSTM layer was designed following a dropout layer (0.5) on the fully connected layer. Finally, for binary classification, we used the softmax activation function to specify the outputs. The architecture can be used in combination with augmentation techniques to artificially expand a dataset by creating new samples that are variations of the original samples. In the context of Raman spectra processing, augmentation can be used to generate new samples that have different frequencies and different Raman shift intensities to increase the size of the dataset and improve the robustness of a classifier. We apply augmentation at the window level on the raw spectra before windowing.

The first kind of augmentation is to increase or reduce the Raman shift intensity. This involves randomly modifying the Raman shift intensities of a window by a small amount. The shift amount is usually determined by a uniform or normal distribution with a small mean and standard deviation. In this way, new spectra can be generated that are similar to the original but have slight differences in the intensity of the peaks (10% in this study). This can help the classifier learn to be more robust to variations in the Raman shift axis that may occur due to instrument calibration, sample preparation, or other factors.

The other augmentation applied in this study is frequency variation. In this case, the frequency axis of a window is randomly shifted by a small amount. Again, the shift factor is typically determined by a uniform or normal distribution with a small mean and standard deviation. In this way, new spectra can be generated that are similar to the original but with slight variations (10% in this study) in the position of the peaks within the window. This can help the classifier learn to be more robust to variations in the peak spectral range that may occur, for example, due to recalibration of the system.

Both augmentation techniques can be applied independently or together to create a larger and more diverse dataset for training a classifier. In this study, we performed an ablation study in which we applied both techniques independently and together to investigate the effects on the resulting performance of the classifier.

However, it is important to note that the amount and type of augmentation used should be carefully chosen to avoid overfitting the model to the augmented data, which can lead to poor performance on real-world data. In addition, it is important to validate the performance of the classifier on a separate test set containing real-world spectra that were not used for training or augmentation. For this reason, both the validation and test data are never augmented in our study.

When misclassifications can have serious consequences, such as in medical diagnoses, it is essential to ensure that the classifier’s predictions are accurate and reliable. One approach to improving the reliability of a CNN-based classifier is to provide a reject option. With a reject option, the classifier can indicate when it is uncertain about its predictions and when it cannot confidently classify a cell. Instead of forcing a decision, the classifier can reject the cell and request further samples. Introducing a reject option in the proposed LSTM-CNN-based classifier can have a significant impact on its performance, as rejecting cells can reduce the number of false positive classifications, resulting in higher precision. On the other hand, rejecting too many cells can result in even lower recall thus reducing the overall accuracy. By adjusting the decision threshold, it is possible to balance the trade-off between precision and recall and maximize overall accuracy. In this study, we therefore introduced two levels of reject options to test whether they have a positive impact on the actual classifier performance resulting from Hyper-parameter optimization.

To implement a reject option, an architecture based on SelectiveNet [29] is adopted. SelectiveNet adds a branch to the network alongside the main prediction branch. This additional branch is responsible for estimating the confidence or uncertainty of the main branch’s predictions. If the uncertainty surpasses a certain threshold, the network rejects the prediction. The selective branch can be implemented in various ways, such as a separate neural network, an auxiliary CNN, or a few additional layers in parallel with the main branch. We opted, as shown in [29], to add a few additional layers in parallel with the main branch. This also required a loss function for the SelectiveNet branch that measures the uncertainty or confidence of the predictions. A thresholding mechanism to determine when to reject a prediction has been implemented. If the confidence score is below this threshold, the network rejects the prediction. Adding the reject option can improve the reliability of predictions in situations where the network is uncertain, improving the precision. In this work, two thresholds have been compared during assessment (refer to Section 3.6 for adopted hyper-parameters ranges).

#### 2.4.4. Blind Prediction

To evaluate the predictive ability of the developed artificial intelligence models, we performed a blind prediction on unknown spectra, as schematically shown in Figure 1e, not used in model definition. In particular, we selected three sets of cells composed of different percentages of Tumor cells (namely, MIX sets).

## 3. Results and Discussion

### 3.1. Morphological Analysis

**Figure 2:**
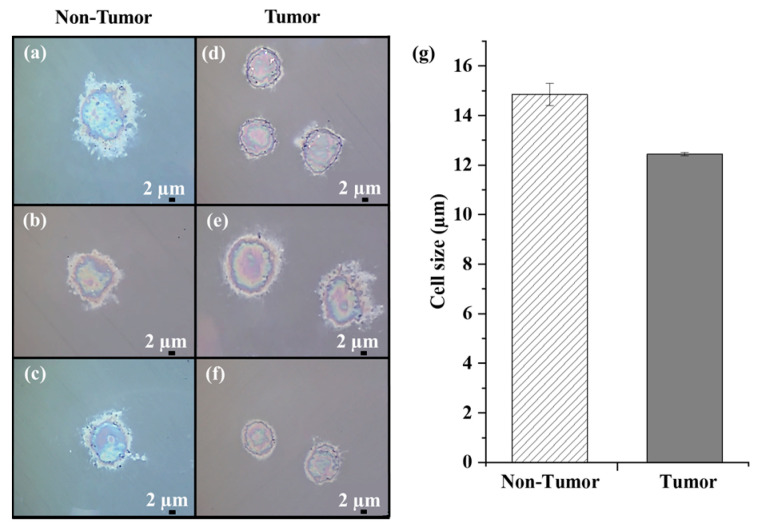
Bright-field microscope images of uncultured human Non-Tumor (**a**–**c**) and Tumor cells (**d**–**f**) fixed on CaF_2_ slides (100× magnification; scale bar 2 µm). (**g**) Dimensional analysis of all Non-Tumor and Tumor cells.

Primary liver cells were obtained from resected HCC tumor tissue and their adjacent non-tumor counterpart. A preliminary morphological analysis was performed by bright-field microscopy. Representative images of uncultured Non-Tumor and Tumor cells are shown in Figure 2.

Differences in the morphological characteristics of the two samples can be appreciated. Hepatocytes exhibited polyhedral or round shapes and a central round nucleus (Figure 2a–c). Morphologically, HCC cells showed variable shapes (mostly irregular or round), large nuclei, and increased nuclear/cytoplasmic ratio (Figure 2d–f), consistent with their altered and heterogeneous phenotypic characteristics.

We estimated the average cell size of the Non-Tumor and Tumor cell samples and found a value of approximately 15 µm and 12.4 µm, respectively. To note, the primary HCC cells were smaller than their non-tumor counterpart (Figure 2g), in accordance with the different sizes exhibited by cancer cells compared to non-tumor cells [30].

### 3.2. Raman Spectroscopy BIOCHEMICAL Overview

The study of the complexity of biomolecular changes occurring between primary Non-Tumor and Tumor cells was performed using RS. For Raman analysis, we analyzed 40 primary liver cells derived from resected HCC tumor tissue and 40 from the adjacent non-tumor area (namely, “Non-Tumor” cells). Specifically, Raman spectra were acquired at five different locations of the nucleus of each cell to account for the heterogeneous distribution of cellular components. A total of 200 spectra were acquired for both samples in the 600–1800 cm^−1^ spectral fingerprint region, which contains the most informative cellular peaks.

**Figure 3:**
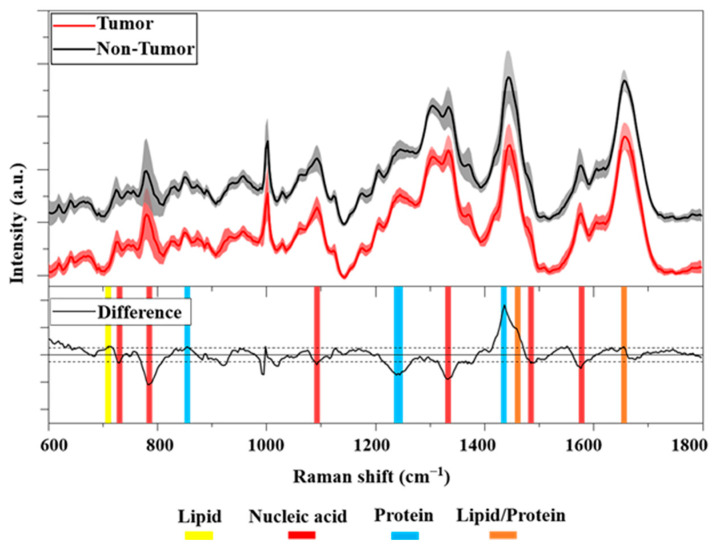
(**Top**): Averaged Raman spectra of Tumor (red line) and Non-Tumor (black line, offset 10%) cells in the FP region. Solid lines show the average over 180 spectra ± standard deviation (shaded areas). (**Bottom**): Difference between averaged Raman spectra (black line). The horizontal solid line corresponds to 0 intensity, the two horizontal dashed lines correspond to a threshold of ±0.025 intensity. Highlighted in yellow, red, and blue are the Raman bands associated with lipids, nucleic acids, and proteins, respectively. In orange, the Raman peaks associated with lipids/proteins are shown.

The characteristic average Raman spectra of Non-Tumor and HCC cells are shown in Figure 3 (top). Both average Raman spectra show a similar spectral pattern. A detailed band assignment of the vibrational modes of the major biomolecules (nucleic acids, proteins, lipids, and carbohydrates) characterizing the cells was summarized in Table 1. In particular, both mean spectra had distinct Raman bands at 782, 1094, 1335, 1370, and 1578 cm^−1^, corresponding to the vibrational modes of the nucleic acid. Other intense peaks were identified at 1004 and 1246 cm^−1^, corresponding to the phenylalanine and Amide III bands, respectively. A prominent peak at 1444 cm^−1^, related to proteins/lipids vibrational bonds, and an intense Amide I band at 1656 cm^−1^ were also evident.

To better illustrate the different biochemical compositions between the cell classes considered, we show in Figure 3 (bottom) the difference between primary Tumor and Non-Tumor cells. The analysis revealed an important difference between the cell samples. In particular, an interesting difference was observed resulting from the nucleic acid at 785 cm^−1^, namely, the negative peak belonged to HCC cells. In addition, some other bands at 1094, 1335, and 1578 cm^−1^ indicate a greater proportion of DNA in the nuclei of cancer cells compared to non-cancer cells. Difference analysis also showed a positive peak at 1438 cm^−1^ belonging to non-cancer cells. In detail, this Raman band was assigned to CH_2_ and CH_3_ deformations in normal tissue [40]. In addition, we also found a negative Raman peak at 1240 cm^−1^ in the spectral region of 1230–1280 cm^−1^ associated with Amide III. Overall, the results showed important spectral differences indicating the higher presence of nucleic acid in the nuclei of cancer cells than in non-cancer cells. This is broadly consistent with other studies in the literature. Notably, some authors reported that the peaks assigned to nucleic acids around 782, 1094, 1335, and 1578 cm^−1^ were mostly assigned to DNA [14,35,38,42]. Many studies have confirmed the association between cancer and an aberrant DNA amount [45,46]. In particular, abnormalities in the nuclear DNA content of hepatocytes have been described as an important risk factor for their transformation into cancer cells [47]. Moreover, recent work found a significant alteration in the nuclear ploidy of HCC tumors [48]. In particular, a correlation between nuclear ploidy amplification and HCC aggressiveness and poor prognosis was demonstrated, thus discovering a new marker for HCC classification.

### 3.3. Dataset

Raman analysis was performed on 40 Non-Tumor and 40 Tumor cells, and 5 Raman spectra were obtained for each cell. After the removal of outliers, the resulting dataset consists of 358 Raman spectra, 181 spectra for Tumor cells, and 177 spectra for Non-Tumor cells, with 19 and 23 spectra, respectively, considered outliers.

### 3.4. Unsupervised Multivariate Analysis

**Figure 4:**
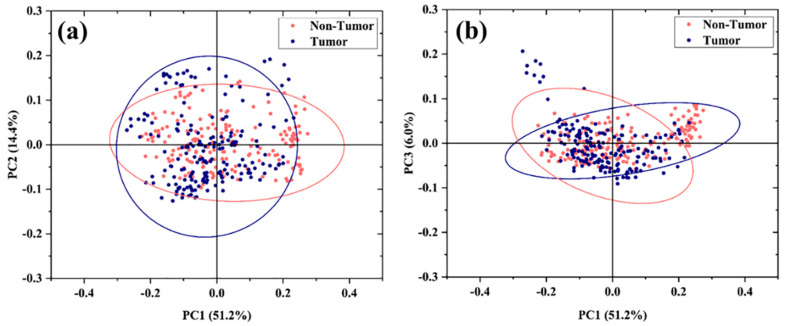
PCA of the Raman spectra. PCA 2D data plot distribution of spectra of uncultured Tumor and Non-Tumor cells based on the first 3 PC components: (**a**) PC1 vs. PC2 and (**b**) PC1 vs. PC3. The ellipses account for a confidential level of 95% of the data.

Principal Component Analysis (PCA) was performed on the entire dataset using the Covariance Matrix. The results are shown in Figure 4a,b. Starting with PC1 (51.2% of the total variance), the following PCs describe differences in the FP region, which account for progressively smaller proportions of the total variance. Interestingly, the loading plot of PC1 (see Figure S1 in Supporting Information) shows a high similarity with the spectrum obtained by subtracting the mean spectra of Tumor and Non-Tumor, suggesting that PC1 explains this main difference. The first three PCs, accounting for 71.6% of the cumulative variance, are used to build the related 2D scatter plots. As can be seen, the data distribution shows a considerable overlap of the data.

The difficulties in discrimination by PCA analysis can be explained by the intrinsic intra- and intercellular heterogeneity characteristic of primary cells. Indeed, tumors are complex tissues consisting of a heterogeneous cell population composed of the coexistence of Tumor and Non-Tumor cells as well as other cell types (stromal, endothelial, and immune cells) [49]. This characteristic cellular heterogeneity is also manifested at the level of single-cell and cell populations [50].

It is worth mentioning that many scientific studies frequently use cultured primary hepatocytes as in vitro models for HCC [51]. However, cultured cell lines may differ from in vivo Tumor cells just by their homogeneity and could lead to misleading results in relation to the mentioned heterogeneity of primary cells [49]. As an example, in the Supporting Information, we show PCA analysis of a few (five) primary cells compared with the corresponding cultured tumor cells (see Figure S2). The 2D scattering plot clearly shows that the cultured cells can have a high degree of similarity, which is due to the artificial growth conditions of the cultured cells, while the corresponding primary Tumor cells can be clustered separately. Therefore, to assess the extent to which Raman spectroscopy can identify Non-Tumor cells and Tumor cells in a realistic scenario, we worked exclusively with primary cells. Considering that PCA alone is not able to fully highlight such subtle differences, in the following, we explore different approaches of Supervised Multivariate Analysis to extract the most useful features for distinguishing experimental groups and classifying spectra.

### 3.5. Supervised Multivariate Analysis

To discriminate the data and maximize the variance between the two groups, LDA was applied as a supervised multivariate analysis directly on Raman spectra. The model was built using the pre-processed data after outliers were removed.

To evaluate the accuracy of the LDA model, we used a training test split procedure to generate a random split of 80% training set and 20% test set. The prediction accuracy of the LDA model was estimated to be 77% (see confusion matrix in Table S1 in the Supporting Information).

**Figure 5:**
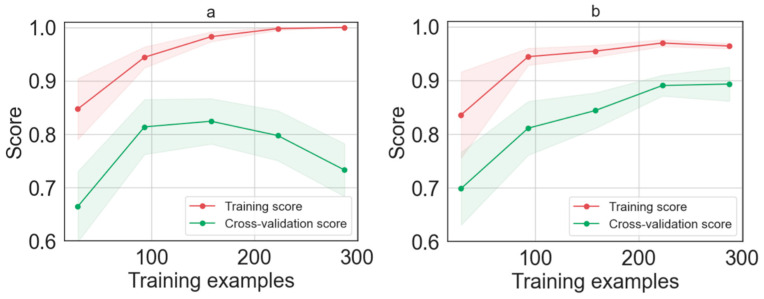
Learning curve as function of the number of training examples for (**a**) LDA model and (**b**) LDA model after Hyper-parameters tuned the optimization methods.

To understand the reason for the low prediction accuracy, we examine the bias–variance trade-off of the model by plotting the learning model in Figure 5a. Ideally, the higher the number of training samples, the better the prediction performance of the model would be. However, as can be seen in Figure 5a, the trend of the learning model shows an excessive maximization of the training accuracy, while it is not able to generalize well from the training data to the test data. This effect is known as overfitting and may be related to the quality of the training dataset and, in particular, to the presence of noise in the training data. Indeed, an overfitted model tends to include all features, even those that have a very limited effect or may worsen the final classification. To reduce the impact of overfitting and improve the model accuracy, we optimized the machine learning method using a “Hyper-parameter” optimization. We integrated, as an optimization algorithm, the contracting grid search method to automate the tuning of the Hyper-parameter. In the grid search, starting from a finite set of possible values for each Hyper-parameter, an exhaustive search for the optimal values is performed using k-fold cross-validation [27]. The result is an optimized LDA model that maximizes the weight of the useful features and minimizes the weight of the useless ones.

Figure 5b shows the learning curve after applying the grid search optimization. The result shows that tuning the Hyper-parameters using the grid search improves the LDA model. Indeed, it can be seen that, after optimization, the error rate decreases in both training and cross-validation until it reaches a stationary value. Specifically, when the learning curve achieves 225 spectra (45 cells), the performance of the model is saturated. Adding more data does not lead to a significant increase in performance. The optimized LDA model was validated on the 20% test set, that is, we used a training dataset accounting for 320 spectra (64 cells), validated using 80 spectra (16 cells). Since we tested our model with a number of samples much larger than the minimum dataset required by the learning curve, we can consider our dataset sufficiently large.

To determine the number of correct classifications and misclassifications when the optimized LDA model makes predictions for each primary liver cell class, we report the confusion matrix in Table 2 for the known test set (Non-Tumor = 37 and Tumor = 36). The accuracy was calculated to be 89%. In addition, the classification performance of the model was calculated in terms of sensitivity, specificity, and precision (see Table S2 in Supporting Information). Despite the high similarity between the Raman spectra of Tumor and Non-Tumor cells, the optimized LDA model provides very high accuracy (approximately 89%).

As further confirmation, we developed another machine learning model exploiting the PC components. We used PCA to reduce the dimensionality of the starting dataset and select only the features that are useful for the model, namely only the PC components that account for a higher percentage of the total variance. The selected PC components were then used to feed the LDA model. The selection of the optimal number of PC components is not trivial, since a smaller number of components can cause the loss of important features, while a larger number of PC components would not solve the overfitting issue.

Accordingly, an LOOCV for the classification model was performed using different numbers of PCs from 5 to 60, and an optimum value of 30 PC components was selected, accounting for 94.3% of the cumulative variance and discharging the PC components accounting for less than 0.085% of the total variance (see Figure S3 and Table S3 in Supporting Information file).

### 3.6. CNN-Based Classification (Supervised Learning)

In the neural models based on the CNN-LSTM network shown in Figure 1, the training phase is applied directly to the Raman spectra, which are partitioned into fixed-size windows. The training phase also includes the Hyper-parameter optimization phase, which had the task of determining:

- The optimal number of layers, which we chose to be between 22 and 56.
- The most appropriate learning rate in the interval [0.0001,0.05].
- The best dropout chosen in the interval [0.1,0.25].
- The threshold of the reject option (in the interval {0.4, 0.8}).
- The type of augmentation to apply to the training set (selected from the set {No augmentation, Frequency, Value, Both}).
- The windows are chosen in order to obtain k_s_ = 3 with no overlap.
- A cross-validation approach with k = 5 folds is used.

**Figure 6:**
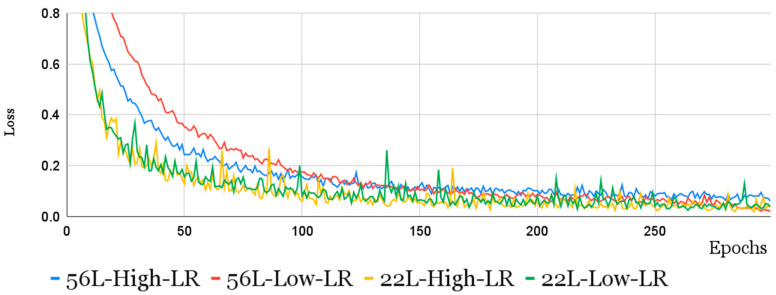
The loss function trend over epochs, during training, for CNN-LSTM models of 22 and 56 layers with different learning rates.

The results of the CNN-LSTM-trained model are shown in Table 3. As can be seen from the table, the absolute best results for the largest network are obtained by applying a lower learning rate. This naturally also leads to a longer training time. However, it is interesting to note that the network with 25 layers, although achieving lower performance in the best case, is more competitive, exceeding the 0.8 F1-score for much lower epoch values than the network with 56 layers (≈80 epochs compared to ≈250), clearly indicating a trade-off, between training times and maximum performance, to be considered in this context. This is also confirmed by the trend of the loss function when epochs vary during training. This trend, shown in Figure 6, clearly indicates that as the number of layers increases and the learning rate decreases, the model requires a greater number of epochs to achieve the same level of performance. It is also interesting to analyze the impact of augmentation and reject options.

From Table 3, it can be seen that the greatest effect is obtained by applying both types of augmentations, since individually, they have little effect on the results. On the other hand, applying both types together allows the classifier to be more robust and to generalize better to real samples that have never been submitted to the network. This result is consistent for both the 25 and 56 models, but the impact is lower for the larger net.

As regards the reject option, the best threshold value is equal to 0.8 (high threshold). In this case, consistently and regardless of the use of the augmentation, the impact of the reject option is equal to ≈3% of the overall performance (F1-score).

In summary, compared with the PCA-LDA approach, the CNN-LSTM model achieves a slightly better F1 score but requires a higher training effort.

This is also confirmed by the confusion matrix presented in Table 4, which shows that the best neural model outperforms the best-tuned PCA-LDA model for both true positives and false positives being equivalent to false negatives.

### 3.7. Blind Prediction of Tumor Cells

In order to assess the performances of the developed artificial intelligence models, we prepared and analyzed cells with different ratios of Tumor to Non-Tumor cells and compared the obtained results with their nominal percentages. Specifically, we analyzed samples with the following ratios of Tumor to Non-Tumor cells: 5 to 0, 4 to 1, and 3 to 2. Raman spectra were acquired, pre-processed, and classified with the same setup instrument, procedures, and parameters used to build the training model.

Table 5 shows that, for the sample of 45 spectra containing only tumor cells, we correctly classified 40 and 39 spectra, with a percentage of correct predictions at the single-spectrum level of 89% and 87% for PCA-LDA and Hyper-parameter-tuned LDA, respectively. For samples with a 4-to-1 Tumor/Non-Tumor cell ratio with a total number of 136 spectra, the percentages of spectra classified as tumors are 82% and 80% for PCA-LDA and Hyper-parameter-tuned LDA, respectively. For the sample with a 3-to-2 Tumor/Non-tumor cell ratio with a total number of 93 spectra, the percentages are 58% and 62% for PCA-LDA and Hyper-parameter-tuned LDA, respectively. These results confirm the predictive ability of the two models.

As for the CNN-LSTM models, the last two rows in Table 5 give the results for the three blind sets, as was performed for the PCA-LDA model. As can be seen from the table, all models trained using the Raman spectra are able to satisfactorily identify the proposed cell mixtures. LDA proved to be particularly effective for MIX1, where 80% of the samples are cancerous, while for the other two mixes, the 56-layer CNN-LSTM network gave a result significantly closer to the nominal values. It is worth noting that the performance difference between the best 25-layer and 56-layer neural networks is small, which can guide the choice in contexts with limited computer resources.

## 4. Conclusions and Discussion

In this work, we analyzed uncultured primary human liver cancer by Raman spectroscopy. Specifically, 40 primary liver cells derived from resected HCC tumor tissue and 40 coming from the adjacent area of the HCC lesion were analyzed. Preliminarily, the cells were analyzed morphologically and spectrally. Morphological differences in shape and size were observed. In addition, the Raman spectra obtained provide detailed biochemical information about the studied sample, without the need for sample preparation. Indeed, key spectral differences were found, revealing a higher presence of nucleic acid in the nuclei of cancer cells than in non-cancer cells. Unsupervised Multivariate Analysis using principal components was performed, but clear differentiation of cells was not possible using these simple methods. Therefore, specific artificially intelligent routines were developed to analyze the same cells. Both Hyper-parameter-tuned LDA and PCA-LDA methods were used to classify cells and make blind predictions for further cell datasets. An accuracy of nearly 90% was obtained for PCA-LDA. In addition, a neural-based approach integrating Convolutional and Recurrent neural networks (i.e., a CNN-LSTM model) was chosen to investigate whether this type of neural network is more suitable for this task. Two types of augmentation were used to create a larger and more diverse dataset for training the classifier. Indeed, additional Raman spectra were added to the training dataset by randomly modifying the Raman shift intensities and frequency axis of the spectral window by a small amount. An accuracy of 93% was obtained for the best CNN-LSTM with 56 layers. Further improvements in the classifier robustness can be envisaged by exploiting additional augmentation strategies. Specifically, more sophisticated data augmentation techniques beyond simple intensity and frequency variation could be investigated. For instance, future research should consider applying domain-specific augmentation methods, such as simulating instrument noise, sample impurities, or other common sources of variability in Raman spectra based on domain expert knowledge. These techniques can make the classifier even more robust in a real-world scenario, where the Raman data are collected by different instruments in different laboratories.

Overall, the reported results clearly demonstrate the effectiveness of AI-assisted Raman spectroscopy in analyzing HCC primary cells for Tumor cell classification and prediction with an accuracy of nearly 90%. Perhaps the main limitation of our analysis pertains to the number of patients since we investigated cells coming from a single patient. Currently, further studies are underway to expand the training dataset and generalize the model. Nonetheless, the promising results herein reported can set the basis for further investigations and pave the way for a wider clinical study. Indeed, we have shown that the synergy between Raman spectroscopy and machine learning in primary cancer cells can be used to discriminate between Tumor and Non-Tumor cells from liver tissues. This approach could provide an effective analytical tool for cancer diagnosis and rapid intraoperative classification.

The impact of AI-assisted Raman spectroscopy to recognize primary human liver cancer cells has manifold potential clinical applications.

The more straightforward application consists of in vitro cytological studies by AI-assisted Raman spectroscopy as an alternative methodology to provide fast analysis of cells under test. Alternatively, AI-assisted Raman spectroscopy can be used for ex vivo studies of resected tissues [18] by exploiting compact Raman systems [52,53].

Basically, in both cases, we envisage the possibility of creating a large database (a clinical bank) collecting anonymized Raman spectra of cytological or histological samples, which, exploiting the rich biochemical information underlying Raman spectra, can provide unattended correlations and useful information for disease diagnosis and prognosis. Finally, commercial Raman probes [54,55] as well as innovative optical fiber probes integrated into fine-needle aspiration biopsies [56] could be used as powerful tools to translate such technology in in vivo clinical scenarios.

## Figures and Tables

**Table 1:** Assignment of characteristic Raman bands of Tumor and Non-Tumor cells.

Raman Shift (cm^−1^)	Assignment	Biomolecule
729	A ring br.	Nucleic acid [31]
757	Trp ring br.	Protein [32]
782–785	DNA backbone, U, C, T ring br.	Nucleic acid [14]
826	O–P–O str.	Nucleic acid [33]
854	Tyr ring br.	Protein [31]
840–860	Polysaccaride structure	Carbohydrates [34]
1004	Phe ring br.	Protein [35]
1031	Phe C–H in-plane bend.	Protein [33]
1064	Skeletal C–C str.	Lipids [33]
1094	Sym. PO_2_^-^ str.	Nucleic acid [35]
1177	Tyr C-H bend.	Protein [31]
1207	Phe, Trp C–C_6_H_5_ str.	Protein [36]
1240–1246	Amide III	Protein [33]
1305	(CH_2_) twist.	Lipids/Protein [37]
1335	A, G ring br., C–H def.	Nucleic acid/Protein [38]
1370	DNA bases ring br.	Nucleic acid [39]
1438	CH_2_, CH_3_ def.	Protein [40]
1444	CH_2_, CH_3_ def.	Lipids/Protein [41]
1578	A, G ring br.	Nucleic acid [42]
1606	Tyr, Phe C=C bend., C NH_2_	Protein/Nucleic acid [43]
1618	Phe, Tyr and Trp C=C	Protein [44]
1656	C=C str., Amide I	Lipids/Protein [31]

br. = breathing mode, str. = stretching mode, bend. = bending mode, def. = deformation mode, twist. = twisting mode, sym. = symmetric, Phe = phenylalanine, Trp = tryptofan, Tyr = tyrosine, A = adenine, U = uracil, C = cytosine, T = thymine.

**Table 2:** Confusion matrix of the Hyper-parameter-tuned LDA models.

Confusion Matrix (Hyper-Parameter LDA)			
Predicted	Non-Tumor	31 (93.94%)	6 (15.00%)
	Tumor	2 (6.06%)	34 (85.00%)
		Non-Tumor	Tumor
		True	

**Table 3:** Training and test results for the CNN-based classifier with different Hyper-parameters, reject options, and augmentation strategies.

# Layers	LR	Dropout	Reject Option	Augmentation	Prec	Rec	F1
			LOW (0.2)	NO	0.816	0.816	0.816
			LOW (0.2)	YES (freq)	0.822	0.835	0.828
			LOW (0.2)	YES (value)	0.832	0.815	0.823
25	0.001	0.15	LOW (0.2)	YES (both)	0.872	0.872	0.872
			HIGH (0.8)	NO	0.825	0.818	0.821
			HIGH (0.8)	YES (freq)	0.834	0.838	0.836
			HIGH (0.8)	YES (value)	0.847	0.848	0.847
			HIGH (0.8)	YES (both)	0.899	0.899	0.899
			LOW (0.2)	NO	0.826	0.826	0.826
			LOW (0.2)	YES (freq)	0.843	0.848	0.845
			LOW (0.2)	YES (value)	0.865	0.877	0.871
56	0.01	0.2	LOW (0.2)	YES (both)	0.899	0.899	0.899
			HIGH (0.8)	NO	0.902	0.921	0.911
			HIGH (0.8)	YES (freq)	0.912	0.904	0.908
			HIGH (0.8)	YES (value)	0.912	0.915	0.913
			HIGH (0.8)	YES (both)	**0.943**	**0.917**	**0.930**

**Table 4:** Confusion matrix of the best-tuned LSTM-CNN model (last row of Table 3).

Confusion Matrix (LSTM-CNN)			
Predicted	Non-Tumor	33 (91.67%)	2 (5.40%)
	Tumor	3 (8.33%)	35 (94.59%)
		Non-Tumor	Tumor
		True	

**Table 5:** Classification of samples with different ratios of Tumor and Non-Tumor cells.

	Tum (% Tum)	MIX1 (% Tum)	MIX2 (% Tum)
Nominal value	100.00%	80.00%	60.00%
Hyper-parameter tuned LDA	87.00%	**80.10%**	62.30%
PCA-LDA	89.00%	82.40%	58.00%
CNN-LSTM-22	91.60%	82.76%	58.33%
CNN-LSTM-56	**92.70%**	81.67%	**61.54%**

## Data Availability

The dataset and the AI codes (Hyper-LDA and CNN) can be found on GitHub at the following link: https://github.com/unisannio-phd-ite/liver-cancer-detection-using-raman-spectroscopy, accessed on 5 November 2023.

## Supplementary Materials

The following supporting information can be downloaded at: https://www.mdpi.com/article/10.3390/cells12222645/s1, Figure S1: Loading Plot of principal component 1 (PC1); Figure S2: PCA plot associated with the spectra of five uncultured and cultured Tumor cells. Figure S3: Leave-one-out-cross—validation (LOOCV) as a function of the number of PCs. Table S1: Confusion matrix of the LDA model; Table S2: Optimized LDA classification metrics; Table S3: List of the first 35 PC components and the relative percentages of variance. Public Repository for results reproducibility.
