# Supplementary material for: Assessment of Primary Human Liver Cancer Cells by Artificial Intelligence-Assisted Raman Spectroscopy
Source: Cells. 2023 Nov 17;12(22):2645. doi: 10.3390/cells12222645 (PMC10670489; doi:10.3390/cells12222645)
Supplement: Supplementary file 1 [file cells-12-02645-s001.zip › cells-2648868-supplementary.pdf]

## S1. Metrics

Prediction accuracy (Acc) is defined as the proportion of correct predictions to the total number of predictions, can be calculated as follows:

$$Acc = \frac{(TP+TN)}{(TP+TN+FP+FN)}, \quad \text{(S1)}$$

where true positives (TP) are correctly labelled as positive, false positives (FP) are incorrectly labelled as positive, true negatives (TN) are correctly labelled as negative, and false negatives (FN) are incorrectly labelled as negative.

Sensitivity (SNC): Refers to the actual true positive the model was able to predict and can be explained as

$$SNC = \frac{TP}{TP+FN}, \quad \text{(S2)}$$

Specificity (SPC): refers to the actual true negative the model was able to predict and can be written as:

$$SPC = \frac{TN}{TN+FP}, \quad \text{(S3)}$$

Precision (PRC): out of all predicted Positive cases, how many were actually Positive", or

$$PRC = \frac{TP}{TP+FP}, \quad \text{(S4)}$$

Finally, F1-Score is a combination of the precision and recall of the model by harmonic mean:

$$F1 = \frac{2*TP}{2*TP+FN+FP}, \quad \text{(S5)}$$

## S2. Figures

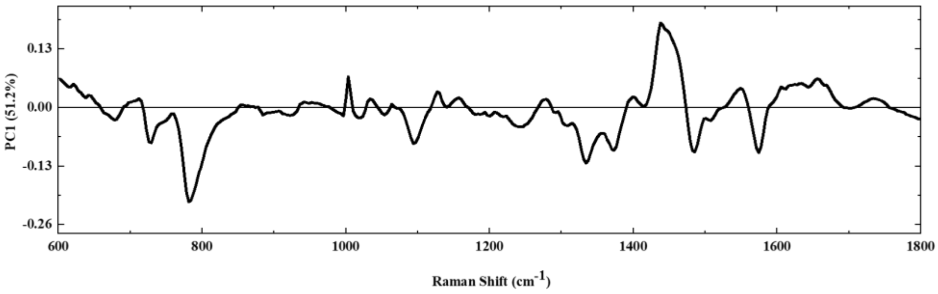


**Figure S1.** Loading Plot of principal component 1 (PC1).

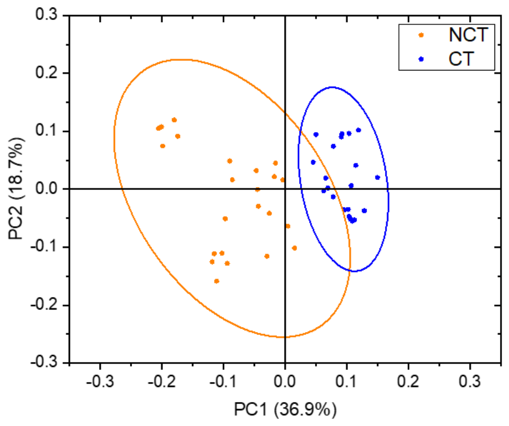


**Figure S2.** PCA plot associated to the spectra of five uncultured and cultured Tumor cells. The 2D plot is based on the first 2 PCs components: PC1 vs PC2.

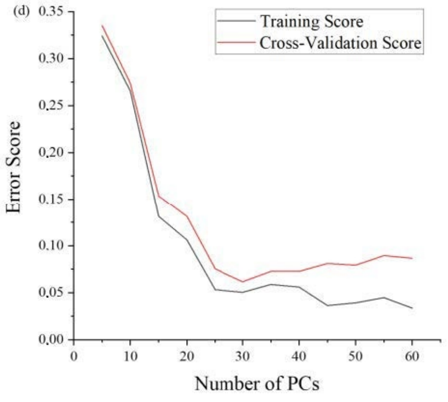


**Figure S3.** Leave-one-out-cross- validation (LOOCV) as a function of the number of PCs.

## S3. Tables

**Table S1.** Confusion matrix of the LDA model.

| Confusion matrix (Hyper-LDA) | | | |
|---|---|---|---|
| **Predicted** | **Non-Tumor** | **24 (85.71%)** | **13 (28.89%)** |
| | **Tumor** | **4 (14.29%)** | **32 (71.11%)** |
| | | **Non-Tumor** | **Tumor** |
| | | **True** | |

**Table S2.** Optimized LDA classification metrics.

| Merit | Scores (%) |
|---|---|
| **Sensitivity** | **94.4** |
| **specificity** | **84** |
| **Precision** | **85** |
| **F1-score** | **89** |

**Table S3.** List of the first 35 PC component and the relative percentages of variance.

| Principal Component Number | Percentage of Variance (%) | Cumulative (%) |
|---|---|---|
| 1 | 51.284 | 51.284 |
| 2 | 14.309 | 65.593 |
| 3 | 6.017 | 71.610 |
| 4 | 5.044 | 76.654 |
| 5 | 4.097 | 80.750 |
| 6 | 2.442 | 83.193 |
| 7 | 2.282 | 85.475 |
| 8 | 1.677 | 87.152 |
| 9 | 1.198 | 88.350 |
| 10 | 0.869 | 89.219 |
| 11 | 0.749 | 89.968 |
| 12 | 0.683 | 90.651 |
| 13 | 0.518 | 91.169 |
| 14 | 0.504 | 91.673 |
| 15 | 0.308 | 91.981 |
| 16 | 0.289 | 92.270 |
| 17 | 0.245 | 92.515 |
| 18 | 0.232 | 92.747 |
| 19 | 0.199 | 92.946 |
| 20 | 0.170 | 93.117 |
| 21 | 0.166 | 93.283 |
| 22 | 0.146 | 93.429 |
| 23 | 0.134 | 93.562 |
| 24 | 0.123 | 93.686 |
| 25 | 0.116 | 93.802 |
| 26 | 0.106 | 93.908 |
| 27 | 0.096 | 94.005 |
| 28 | 0.094 | 94.098 |
| 29 | 0.087 | 94.185 |
| 30 | 0.087 | 94.272 |
| 31 | 0.085 | 94.356 |
| 32 | 0.083 | 94.439 |
| 33 | 0.079 | 94.519 |
| 34 | 0.077 | 94.596 |
| 35 | 0.076 | 94.671 |

## S4. Public Repository for results reproducibility

The dataset and the AI codes (Hyper-LDA and CNN) can be found on GitHub at the following link:
https://github.com/unisannio-phd-ite/liver-cancer-detection-using-raman-spectroscopy

Here we report a detailed description of the PCA-LDA analysis carried out by Origin 2018. In detail, the dataset was first organized in order to have each column representing a sample's spectrum, and each row representing a single frequency. PCA was performed by using the Origin app "Principal Component Analysis for Spectroscopy". The analysis was performed on the entire dataset by using the Covariance Matrix and extracting the maximum number of principal components (PCs), corresponding to the number of spectra used in the analysis.

Once the PCs were obtained, the PCs accounting for a higher percentage of the total variance were selected and used to feed the LDA model.

The settings used for the LDA were the following:

- Prior Probabilities proportional to group size
- Linear Discriminant Function
- Canonical Discriminant Analysis
- LOOCV

To select the optimal number of PCs, a LOOCV for the classification model was performed using different numbers of PCs from 5 to 60. As a result, an optimum value of 30 PC components was selected, accounting for 94.3% of the cumulative variance. Therefore, the PCs accounting for less than 0.085% of the total variance were discharged.
